# Procedural Sedation in a UAE Emergency Department: Encouraging Informed Decision-Making Through a Patient Information Leaflet

**DOI:** 10.7759/cureus.44980

**Published:** 2023-09-10

**Authors:** Amani Khamis AlBedwawi, Afra Bakheet Almansoori, Muna Abdelaziz Aljasmi, Fatema Salem Al Ameri, Nasser Ahmed, Abdul-Salam Adnan Al Mnaseer, Ismail Mohamed Al Ramahi, Kenneth Charles Dittrich, Hasan Qayyum

**Affiliations:** 1 Emergency Department, Sheikh Khalifa Medical City, Abu Dhabi, ARE

**Keywords:** audit, consent, patient information, procedural sedation, documentation

## Abstract

Introduction

Procedural sedation is a common procedure conducted in emergency departments (ED) across the world, which requires patients to receive anesthesia/sedation medication in a controlled environment in order to alleviate pain, anxiety, and suffering, thereby allowing multiple procedures to be completed in a safe and timely manner. We deploy this technique for joint reductions, burns dressings, wound repairs, etc. in our ED. As a large tertiary referral hospital ED, we aimed to benchmark our practice for this high-acuity procedure against international standards. The main objective of our audit was to benchmark our current practice of procedural sedation against international standards from the Royal College of Emergency Medicine (RCEM), United Kingdom, and American College of Emergency Physicians (ACEP) guidelines. As a secondary objective, we aimed to design and implement a multi-lingual procedural sedation leaflet for our patients and their carers.

Methods

A retrospective electronic healthcare records review was conducted from January 2019 to August 2022 following which a convenience sample of 100 patients was selected. Records audited were obtained from the Hospital Quality and Pharmacy departments. We selected patients from the data provided by selecting sedation medication used (ketamine, midazolam, propofol) and frequency documented as 'pre-procedure' (Pre-Proc). We included patients of all age groups who received procedural sedation in the emergency department and excluded inpatient encounters. After reviewing RCEM and ACEP guidance, we studied 14 criteria and standards. A team comprising physicians and hospital interpreters was set up to draft a procedural sedation leaflet. After hospital marketing team approval, these were published in Arabic, English, Urdu, Hindi, Bengali, and Malayalam.

Results

Compliance percentages of the 14 criteria were calculated. A “traffic light” color scheme was used to inform the reader of areas of good practice and areas for improvement. Percentages of 90-100% (green) were considered compliant, 80-89% (amber) were partially compliant, and 79% or less (red) were non-compliant. Of the 14 criteria, 10 were fully compliant. One criterion was partially compliant and three criteria were non-compliant.

Conclusion

Overall, we performed well in in this audit with 100% compliance rates in many areas. We identified that we had no written discharge information leaflet for our patients and carers. We drafted a multi-lingual procedural sedation leaflet and stocked this in the department. Through face-to-face education, we re-trained physicians on the importance of documentation when adhering to safe practices around procedural sedation.

## Introduction

Procedural sedation is a common procedure conducted in emergency departments (ED) across the world, which requires patients to receive anesthesia/sedation medication in a controlled environment to alleviate pain, anxiety, and suffering, thereby allowing multiple procedures to be completed in a safe and timely manner. This technique is deployed for joint reductions, burns dressings, wound repairs, etc.

Differing from general anesthesia, procedural sedation is defined as the administration of one or more pharmacological agents to facilitate a diagnostic or therapeutic procedure while targeting a state during which airway patency, spontaneous respiration, protective airway reflexes, and hemodynamic stability are preserved while alleviating anxiety and pain [1,2]. 

To ensure safe practices of procedural sedation and to identify areas of improvement, it is recommended that EDs regularly audit compliance [2,3]. As a large tertiary referral hospital ED, we aimed to benchmark our practice against international standards. The main objective of our audit was to benchmark our current practice of procedural sedation against criteria based on recommendations from the Royal College of Emergency Medicine (RCEM), United Kingdom, and American College of Emergency Physicians (ACEP) guidelines. As a secondary objective, we aimed to design and implement a multi-lingual procedural sedation information leaflet for our patients and their carers.

## Materials and methods

We reviewed procedural sedation international guidance from the RCEM [3] and the ACEP [4] as well as locally available clinical policy guidelines to identify criteria to audit our practice of procedural sedation. We developed criteria that would apply to our practice in the United Arab Emirates and could be used to improve the quality of care in sedation practices. Table 1 summarises recommendations on procedural sedation in the ED from the RCEM, while Table 2 summarises recommendations from the ACEP Clinical Policies Subcommittee (Writing Committee) on Procedural Sedation and Analgesia.

**Table 1:** Summary of recommendations from the Royal College of Emergency Medicine, United Kingdom, Best Practice Guidelines, Procedural Sedation in the Emergency Department (August 2022)

Recommendations
Every emergency department should have a sedation lead responsible for ensuring the appropriate governance structures are in place in relation to procedural sedation.
Emergency departments undertaking paediatric procedural sedation should have a nominated paediatric sedation lead and specific paediatric guidelines.
The use of a sedation proforma or similar electronic equivalent is strongly recommended.
Processes should be in place for adverse incident reporting arising from procedural sedation as well as rapid investigation of significant events.
Emergency departments should have clear policies with regard to competencies for the provision of procedural sedation in both adults and children as well as, up-to-date lists of those clinicians fulfilling the competencies. Simulation training sessions should be used to promote safe and effective procedural sedation in line with local policies.
Procedural sedation should take place in a designated area of the emergency department with the requisite staffing levels and equipment e.g., resuscitation room.
Procedural sedation should not take place without careful consideration of the analgesic requirement for the procedure, taking into account any analgesics already administered.
The clinician who will be responsible for providing the procedural sedation should undertake a pre-procedure Safety Brief with the other members of the team.
The use of oxygen during procedural sedation is encouraged especially for at-risk patient groups (e.g., ischaemic heart disease) and those undergoing deep sedation procedures (increased risk of short periods of apnoea).
Monitoring during procedural sedation should include: 3 lead ECG, oxygen saturations, continuous capnography, non-invasive blood pressure.
The use of a patient advice leaflet is encouraged.

**Table 2:** American College of Emergency Physicians policy statement, Procedural sedation in the Emergency Department (February 2023)

Guidelines
Emergency physicians who have received the appropriate training and skills necessary to safely provide procedural sedation, such as board certification in emergency medicine or graduates of an ‘Accreditation Council for Graduate Medical Education’(ACGME) accredited emergency medicine program, should be credentialed without additional requirements for procedural sedation.
The decision to provide sedation and the selection of specific pharmacologic agents should be individualized for each patient by the emergency physician and should not be otherwise restricted.
Emergency physicians and staff are expected to be familiar with the pharmaceutical agents they use and be prepared to manage their potential complications.
To minimize complications, the appropriate drugs and dosages must be chosen and administered in an appropriately monitored setting. Patient evaluation should be performed before, during, and after their use.
Institutional and departmental guidelines related to the sedation of patients should include the selection and preparation of patients, informed consent, equipment and monitoring requirements, hospital staff training and competency verification, criteria for discharge, and continuous quality improvement.
ED physician and nursing leadership should have ongoing collaborations to develop institutional policy regarding nursing roles in sedation and the ability of nurses to administer sedatives. Emergency nurses with demonstrated competencies are qualified and capable of safely administering propofol, ketamine, and other sedatives.

We identified 13 criteria as listed in Table 3. Compliance percentages of 90-100% was considered compliant, 80-89% was partially compliant, and compliance of 79% or less were considered non-compliant.

**Table 3:** Our proposed audit criteria with standards set at 100%. Our audit criteria were tailored to meet our requirements after reviewing audit criteria from the Royal College of Emergency Medicine, United Kingdom and the American College of Emergency Physicians.

Number	Criteria	Standard
Criterion 1	Patients undergoing procedural sedation in the ED should have documented evidence of pre-procedural assessment, including prediction of difficulty in airway management	100% of cases
Criterion 2	Patients undergoing procedural sedation in the ED should have documented evidence of drug doses, routes of administrations, and allergies	100% of cases
Criterion 3	There should be documented evidence of the patient’s consent unless lack of mental capacity has been recorded	100% of cases
Criterion 4	Procedural sedation should be conducted only when a crash cart is available for the patient receiving sedation	100% of cases
Criterion 5	All patients receiving procedural sedation require the presence of a physician managing sedation and a physician performing the procedure for which sedation is required	100 % of cases
Criterion 6	All patients receiving procedural sedation require the presence of a nurse	100% of cases
Criterion 7	All patients receiving procedural sedation require vital signs monitoring which should include ALL of the following: 1. Non-Invasive BP, 2. Pulse Oximetry, 3. ECG monitoring, 4. Vital signs monitoring	100% of cases
Criterion 8	Oxygen and oxygen delivery devices should be readily available to administer to the patient until they return to their baseline status	100% of cases
Criterion 9	The sedation provider must be skilled at providing rescue interventions including but not limited to recognition and management of airway obstruction, cardiorespiratory arrest, and anaphylaxis	100% of cases
Criterion 10	Following procedural sedation, patients will only be deemed suitable for discharge if ALL of the following criteria are met: 1. Awake and age-appropriate alertness, 2. Vital signs return to pre-sedation baseline, 3. Absence of any respiratory depression, 4. Written discharge instructions given	100% of cases
Criterion 11	Any complications related to sedation procedure are documented	100% of cases
Criterion 12	All cases of procedural sedation have a completed procedure note documented as part of their electronic healthcare records	100% of cases
Criterion 13	All patients and carers receiving procedural sedation in ED will receive a patient information leaflet with written discharge information	100% of cases

After approval from our quality department at Sheikh Khalifa Medical City, Abu Dhabi, a retrospective electronic healthcare records review was conducted from January 2019 to August 2022, following which a convenience sample of 100 patients in which all required data points were readily available was selected. As this was part of a service evaluation, formal ethics approval was not required. Records audited were obtained from the Hospital Quality and Pharmacy departments. All data was stored on a Microsoft Excel spreadsheet (Microsoft Corporation, Redmond, Washington, United States). Patient identifiers were removed at the time of data pooling, except for their unique study identification number.

We included patients of all age groups who received procedural sedation in the ED and excluded inpatient encounters. A team comprising physicians and hospital interpreters was formed to draft a procedural sedation patient information leaflet. After hospital marketing team approval, these were published in Arabic, English, Urdu, Hindi, Bengali, and Malayalam. 

## Results

Of the 100 cases included in the study, 79 were male and 21 were female. Four were in the age group of 0-5 years, 19 were aged between five and 18 years, and 77 were above 18 years of age. The commonest sedation agent used was a combination of ketamine and propofol in 41% of cases (n=41), followed by ketamine as a single agent in 29% of cases (n = 29). The commonest indication to perform this procedure was joint reduction/fracture manipulation in 57% (n=57) cases. Table 4 lists the demographics, sedation medication, and indications for the use of procedural sedation.

**Table 4:** Patient demographics, sedation medication, and indications for use of procedural sedation.

Variables	Number of patients
Age (years)	
Age < 5	4
Age 5-18	19
Age >18	77
Gender	
Male	79
Female	21
Sedation Medicine(s) Used	
Ketamine	29
Midazolam	3
Propofol	9
Combination of ketamine and midazolam	11
Combination of ketamine and propofol	41
Any Other Combination	7
Indication	
Suturing	27
Joint reduction	59
Burns	5
Facilitating imaging, eg CT	5
Incision and drainage	3
Foley Catheter insertion	1

All patients undergoing procedural sedation in this audit had recorded consent by the patient or their legal guardians in the case of children (aged less than 18 years). All cases were performed in the resuscitation area with readily available access to a crash cart. All cases had oxygen delivered to them as part of the procedure and ventilation was monitored using end-tidal capnography. All patients had vital signs recorded pre- and post-sedation. All cases of procedural sedation had an assigned member of nursing staff responsible for monitoring the patient and where needed, administering medication. There were no complications (hypoxia, agitation, allergy, etc). They were either admitted, transferred, or discharged when they were awake to their baseline level. 

We found that 80% of cases had documented evidence of pre-procedural assessment, including prediction of difficulty in airway management and an American Society of Anesthesiologists (ASA) score. Sixty-six percent of patients undergoing procedural sedation in the ED had documented evidence of drug doses, routes of administration, and allergies, while 98% had a separate procedure note documented as part of their electronic healthcare record. 

ACEP guidelines report a paucity of high-quality evidence on the optimal number of staff required to safely perform procedural sedation and analgesia [5]. Some medium-quality studies suggest there was no difference in adverse events between two physicians-one nurse and one phyisican-one nurse models [5]. An ACEP policy statement on unscheduled procedural sedation suggests a minimum of two personnel for safe sedation: a provider who has overall responsibility for sedation and a monitor whose primary responsibility is continuous patient monitoring and documentation [5].

For this audit, we used the RCEM quality standard, which we felt was more objective. They state procedural sedation requires the allocation of three roles: one sedationist, one procedurist, and one nurse [2,3]. Based on this standard, in our practice, we recorded that 73% of cases had a consultant/specialist perform the sedation and a separate consultant/specialist perform the procedure. 

All international guidelines on standards of care for procedural sedation recommend that written care instructions be given to patients, families, and caregivers [2-7]. We found that although verbal advice may have been given, a written discharge leaflet was not available in our ED. 

As part of our recommendations for improvement, we suggested a few measures. Nursing staff were recommended to ensure the sedation room is checked and restocked at the start of every shift and after use for procedural sedation. We delivered education to our physicians on the importance of documenting ASA classification and a formal airway assessment for all cases of procedural sedation. Physicians were also reminded to review the patients’ history of co-morbidities, with a focus on cardiac and respiratory conditions to deem suitability of an ED procedural sedation. It was recommended providers document objective discharge criteria for patients going home. 

**Figure 1:**
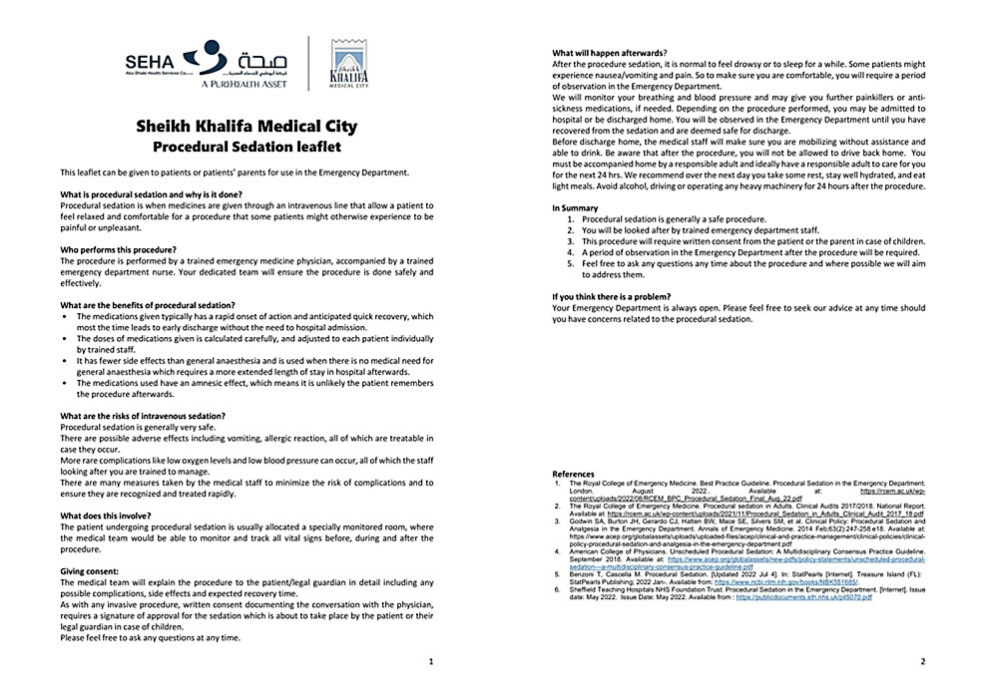
Procedural sedation patient education leaflet (in English)

As stated earlier, a major deficiency of our then practice of sedation was the lack of a written sedation care leaflet for all patients and their carers at discharge after this procedure. Our patient population in Abu Dhabi is diverse and multi-ethnic and as a result, a single-language leaflet would not fit our requirements. We chose to draft patient information leaflets with information on the procedure, the risks/benefits, giving consent, and post-discharge advice in lay terms. This was initially drafted in English (Figure 1). We then incorporated assistance from our ED staff members to translate these into Arabic (Appendix 1), Urdu (Appendix 2), Hindi (Appendix 3), Bengali (Appendix 4), and Malayalam (Appendix 5). After multiple iterations and inputs from our hospital’s marketing team, these were finalized and are now in use in our department. They are provided by staff to patients and their families/accompanying attendants when procedural sedation is being considered.

## Discussion

As stated earlier, procedural sedation is a core skill of an emergency physician. This practice requires physicians to be competent in airway management, ventilation techniques, and cardiovascular sciences. It should be emphasized airway repositioning and bag valve mask ventilation are perhaps the most important airway rescue skills that every ED healthcare worker should be skilled in, including those administering procedural sedation [4]. A sound knowledge of pharmacology is also required [5].

As part of clinical governance, it is recommended all EDs should routinely audit procedural sedation focusing on compliance with the use of sedation proformas, vital signs monitoring during sedation, drugs used for sedation, use of reversal agents, as well as adverse incidents. Standard operating procedures and clinical policies should be in place to encourage safe sedation practices and identify areas of improvement. Incorporating simulation training is recommended to practice safe sedation and associated adverse event drills [2]. 

As in our practice, it is recommended procedural sedation be conducted in a dedicated room in the resuscitation area. It is encouraged that three roles are identified when delivering safe sedation in ED: a separate seditionist, a procedurist, and a nurse at all times [3]. Vital signs monitoring should be used including three-lead electrocardiographic monitoring, pulse oximetry, continuous capnography, and non-invasive blood pressure monitoring [2]. Appropriate oxygen should be readily available and administered to all patients. It is also recommended that a formal assessment for suitability for discharge be conducted and documented [2,3].

Historically, procedural sedation has required a collaborative multi-specialty approach to perform this unique skill with safety, often in an uncontrolled yet pragmatic ED environment. A paradigm shift in this thought process now focuses on physicians and healthcare providers with skill sets and competencies in sedation, rather than specific specialty training [4]. 

It is recommended that the decision to provide procedural sedation be based on the needs of the patient, the risk involved, the benefits conferred, and with due consideration to any alternative non-inferior or superior methods (local/regional anesthesia and non-pharmacological methods). Consent requires an approach of shared decision-making with patients and their carers. This can be as brief or detailed depending on necessity and urgency [4]. Procedural sedation is generally a safe procedure in terms of adverse events. In children sedated using ketamine, mild agitation is common (approximately 20%). Vomiting and hypersalivation have been reported in up to 10% of children. Rare complications like apnoea and laryngospasm occur in less than 1% of cases [8]. In adults, the incidence of hypoxia has been reported to be around 40 cases per 1000 sedations, vomiting in 16 cases per 1000 sedations, hypotension in 15 cases per 1000 sedations, and apnoea in 12.4 cases per 1000 sedations [9].

The seditionist should perform a pre-procedural assessment including a focussed history and examination. It is pertinent to elicit a medication history and allergy history. A previous history of adverse events from anesthesia and/or procedural sedation is mandatory to explore a priori. A pre-sedation airway assessment is strongly recommended to anticipate, plan, and mitigate possible adverse events.

Patients who are healthy or have mild systemic disease (commonly classified as ASA physical status I and II, respectively, are generally excellent procedural sedation candidates. Those with severe systemic disease (commonly classified as ASA III or greater) are at greater risk of adverse events [4,10-12]. A pre-sedation airway assessment is strongly recommended to anticipate, plan, and mitigate possible adverse events. Historically, there have been concerns about fasting and the associated risk of aspiration and other adverse events when applied to procedural sedation. Current practice recommends not to delay procedural sedation in adults or children in ED solely based on fasting time [3,5,13].

It is difficult to postulate a frequency of vital signs where ‘one size fits all.’ We believe ‘vital signs are vital’ and recommend following your local guidance around this to optimize vital signs recording. The highest risk of complications occurs within the first 20 minutes of receiving the sedation medication and during the post-procedure period when external painful stimuli are removed [13,14]. Hence, the presence of an ED nurse till the patient is back to their baseline level of recovery is as important if not more than recording vital signs.

Often due to the unscheduled and unexpected nature of the work in emergency medicine, a clear understanding of the patient's journey is lacking. Regular verbal advice and updates should be provided on management plans. Written advice should also be readily accessible to all patients and their carers transiting through ED [15].

It has been recommended that EDs identify and implement methods of providing and recording the issuance of written discharge advice [2-7]. As this was the largest deficiency in our then practice, we assembled a team of physicians and interpreters who drafted our written patient information first in English, then in Arabic, Hindi, Urdu, Bengali, and Malayalam.

From a clinical governance perspective, the importance of documentation and contemporaneous charting of notes cannot be overemphasized. We recommend having a sedation proforma or a power note on the electronic health record system specific to procedural sedation. The indication of the procedure, correct site identification if applicable, medication and doses administered, allergies, airway assessment, and focused history/exam, start time and end time of the sedation process should all be documented.

## Conclusions

We recommend all EDs should aspire to administer procedural sedation in a safe manner with mechanisms to audit practices in order to check compliance with recommended standards. As part of clinical governance, regular audits should be performed to identify areas of improvement. The provision of a patient information leaflet will serve to allay the concerns of patients and their carers, encourage shared decision-making, and serve as a safety net with clear discharge advice.

## Figures and Tables

**Figure 2:**
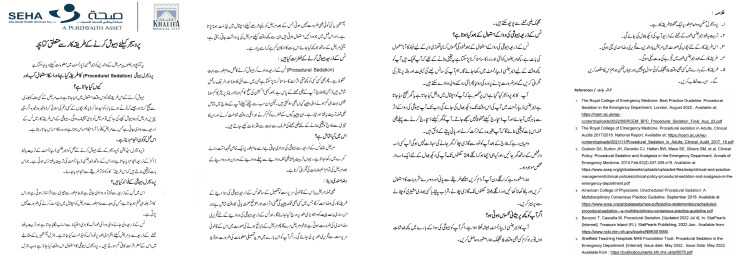
Procedural sedation patient information leaflet (in Arabic)

**Figure 3:**
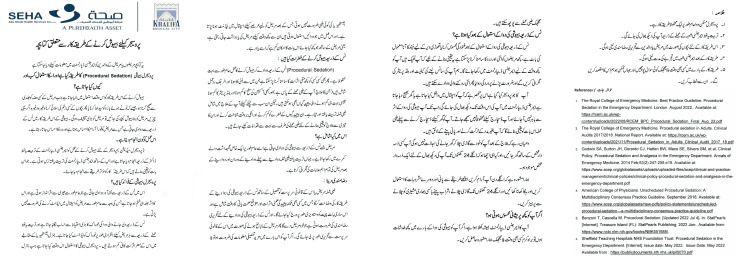
Procedural sedation patient education leaflet (in Urdu)

**Figure 4:**
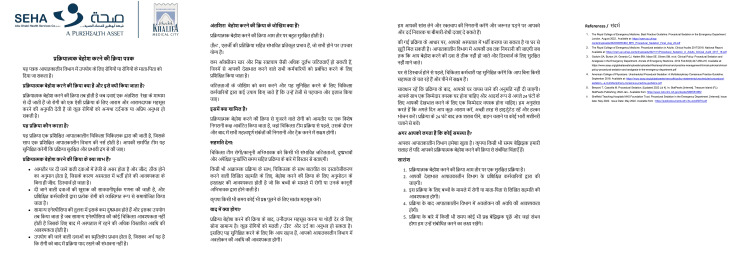
Procedural sedation patient information leaflet (in Hindi)

**Figure 5:**
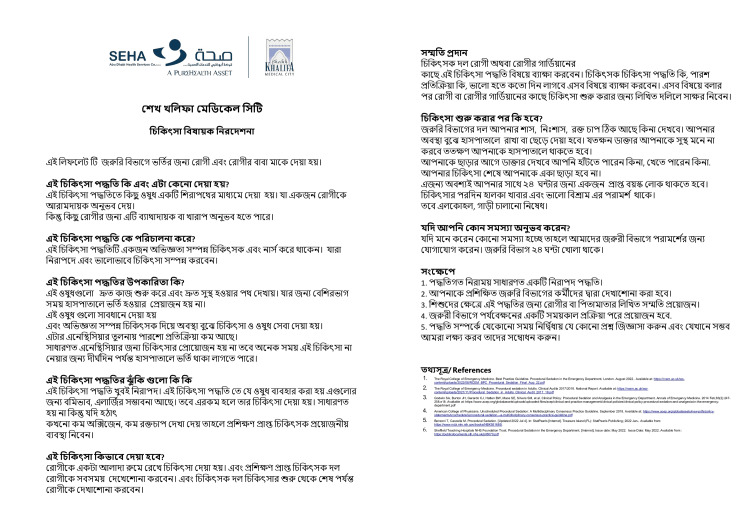
Procedural sedation patient information leaflet (in Bengali)

## Appendices

Appendix 1

 Appendix 2

Appendix 3

Appendix 4
